# A Preliminary Investigation of the Gastrointestinal Bacterial Microbiomes of Barred Owls (*Strix varia*) Admitted to a Wildlife Hospital

**DOI:** 10.3390/ani15111643

**Published:** 2025-06-03

**Authors:** Haerin Rhim, Maria G. Aguilar, Kimberly L. Boykin, Kaylie Zapanta, Janina A. Krumbeck, Mark A. Mitchell

**Affiliations:** 1 Department of Veterinary Clinical Sciences, School of Veterinary Medicine, Louisiana State University, Baton Rouge, LA 70803, USA; magui32@lsu.edu (M.G.A.); mmitchell@lsu.edu (M.A.M.); 2 MiDOG Animal Diagnostics LLC, 14762 Bentley Cir, Tustin, CA 92780, USA; kzapanta@midogtest.com (K.Z.); jkrumbeck@midogtest.com (J.A.K.)

**Keywords:** bird, raptor, wildlife, antibiotics, resistance, rehabilitation, hospitalization, microbiome, microbiota, gut

## Abstract

Research on the gut microbiome, which includes microbial communities and genetic material in the gastrointestinal tract, has revealed essential roles beyond digestion, such as immune regulation, metabolism, and homeostasis. However, studies on birds—key ecosystem members—remain limited. Injured wild birds admitted to wildlife hospitals often receive antibiotics that can alter gut microbiota, leading to dysbiosis and promoting antimicrobial-resistant (AMR) bacteria. This study examined how hospitalization and antibiotics influence the cloacal microbiota of barred owls admitted for fracture repair. A total of 17 cloacal swab samples were analyzed using next-generation sequencing targeting 16S rRNA and AMR genes. Across all samples, Bacillota (Firmicutes), Actinomycetota (Actinobacteria), and Pseudomonadota (Proteobacteria) were the most abundant phyla. In non-antibiotic-treated owls, alpha and beta diversity showed no significant changes between admission and release; however, antibiotic-treated owls exhibited significant diversity shifts in these parameters at release. AMR genes were detected in most samples at admission, with some increasing significantly during hospitalization, suggesting an impact of antibiotic exposure. These findings provide insights into how antibiotics used in wildlife rehabilitation affect host microbiota and contribute to AMR gene dissemination.

## Simple Summary

Birds are vital to ecosystems, yet studies on their gut microbiome remain limited. Wildlife hospitals offer a unique opportunity to study wild birds as environmental sentinels during rehabilitation. Many receive antibiotics, but the impact on their gut bacteria and the potential spread of antibiotic-resistant bacteria after release is unclear. This study examined cloacal samples from barred owls (*Strix varia*), the most commonly admitted species to the Wildlife Hospital of Louisiana. Owls were randomly assigned to antibiotic-treated and non-treated groups, and their cloacal microbiomes were compared at admission and release. A significant shift in the microbiome was detected in antibiotic-treated owls compared to control owls, with a reduction in the richness and evenness of bacteria post-antibiotics. We also detected alterations in antibiotic resistance genes over time, with some acquiring new resistance genes during their hospitalization. These findings demonstrate that rehabilitating wildlife can have a minimal impact on their gastrointestinal microbiome in the absence of antibiotic treatment; however, antibiotic usage can significantly alter the microbiome and potentially increase the risk of translocating antibiotic resistance genes into naïve ecosystems. These findings can be used to guide better antibiotic practices and conservation efforts in wildlife rehabilitation.

## 1. Introduction

Over the past decade, studies on the gut microbiome have recognized that this system serves fundamental roles in maintaining host homeostasis, acting as a reservoir for pathogens, and promoting biodiversity [1,2]. While much of this research has focused on humans, studies on animal microbiomes, including those of over 100 wild avian species, have steadily increased [2]. However, human activities have been shown to significantly disrupt the microbiomes of wildlife, posing serious long-term threats to wildlife populations [3]. These disturbances can lead to biodiversity loss, jeopardizing ecosystem stability and the balance of the biosphere. Wild birds, as essential components of ecosystems, are particularly important in this context. Despite their ecological significance, studies on the microbiomes of wild birds—especially those interacting with humans in settings such as wildlife rehabilitation—remain limited.

Wildlife hospitals play a vital role in treating injured animals and returning them to their natural habitats. For wild birds, fractures and skin injuries caused by negative interactions with humans are among the most commonly observed conditions [4,5]. During treatment, antibiotics are routinely administered to prevent or treat infections. However, antibiotics can disrupt the delicate balance of the gut microbiome, leading to dysbiosis—a state of microbial imbalance characterized by reduced diversity and an abundance of key symbiotic microorganisms. This imbalance can profoundly affect physiological processes, including metabolism, immune responses, and disease susceptibility [6]. For example, studies on chickens (*Gallus gallus domesticus*) have shown that antibiotics can induce significant alterations in the gut microbiota and host metabolism [7]. While extensive research in humans and livestock has highlighted the critical role of gut microbiome dysbiosis in various diseases, these findings have not been widely extended to wild bird species.

In addition to disrupting microbial communities, antibiotic use accelerates the survival and emergence of antimicrobial-resistant (AMR) bacteria, a global issue now regarded as a “silent pandemic” and a major One Health challenge [8,9]. To address this issue, global initiatives such as antibiotic stewardship programs and surveillance efforts have been established to regulate antibiotic use through evidence-based policies and guidelines [10,11]. These strategies emphasize prudent practices aimed at minimizing unnecessary antibiotic use and preserving their effectiveness for the future. One review demonstrated that effective interventions in antibiotic prescriptions successfully reduced excessive usage, leading to a decrease in AMR-related infections and improved clinical outcomes [12]. While some AMR bacteria naturally exist in the environment, their high prevalence in wildlife, even in individuals with no prior antibiotic exposure before admission to rehabilitation facilities, suggests an influence of anthropogenic factors [13,14]. Because rehabilitated animals are released back into the environment, they pose a significant risk as carriers, particularly highly mobile birds, for disseminating resistant bacteria into natural ecosystems.

This pilot study focused on the barred owl (*Strix varia*), the most frequently admitted raptor to the Wildlife Hospital of Louisiana (Baton Rouge, LA, USA), to explore the impacts of rehabilitation practices on its gut microbiome. As an apex predator within the ecosystem’s food chain and a member of the poorly studied raptor family [2], understanding the health and resilience of this species is ecologically significant. Specifically, we aimed to characterize the cloacal (fecal) microbiome of barred owls and assess how antibiotic treatment and hospitalization duration influence microbial diversity and composition. Additionally, we investigated the prevalence of antibiotic resistance genes in barred owls at the time of admission and prior to release back into the wild. By addressing these objectives, this research seeks to provide foundational insights into the effects of rehabilitation practices on the gut microbiomes of wild birds and their role in the dissemination of AMR bacteria.

Most studies characterizing gut microbiota in birds are based on single-sample strategies, with few studies serially evaluating changes related to health status or drug exposure [2]. We hypothesized that the alpha diversity of the gut microbiome in barred owls would be similar at presentation but would differ significantly at the time of release between birds treated and not treated with antibiotics during hospitalization. We also hypothesized that there would be significant differences in beta diversity between the two groups at release. Lastly, we expected to detect AMR genes in barred owls at admission and predicted a non-significant increase in these genes in antibiotic-treated birds over the course of hospitalization.

## 2. Materials and Methods

### 2.1. Animals and Sampling

A prospective, randomized controlled study was performed in accordance with the regulations of the Louisiana State University Institutional Animal Care and Use Committee (23-079). The sample size for this pilot study was determined based on a priori data: an alpha level of 0.05, a power of 0.8, an expected 50% difference in alpha diversity, and a standard deviation of 20% of the mean alpha diversity between groups receiving antibiotics or not. A one-tailed t-test indicated a minimum requirement of 3 birds per group. To reduce the risk of a Type II error, we included all available birds during the study period to further explore microbiome characteristics with a larger sample size.

Seventeen cloacal swab samples were collected from thirteen barred owls with closed fractures that could be repaired with a hybrid tie-in external skeletal fixator and had a good prognosis. These inclusion criteria were selected to ensure the likelihood of release and consecutive samplings. As we opportunistically recruited available birds during the study period, repeated sampling was only achieved in 4 birds. Once admitted, owls received a thorough physical examination, and blood was collected to measure packed cell volume, white blood cell estimates, and total solids. Any individuals showing systemic signs of disease were excluded from the study. Nine birds did not receive any antibiotic treatment (NoAbs), and four birds (Abs) were treated with enrofloxacin (15 mg/kg q 12 h [15]; Bayer, Shawnee Mission, KS, USA) for one to two weeks post-operatively. Treatment duration was based on individual case response and clinician preference. All birds received q 24–48 h cleaning of the pins with dilute betadine during the period that the hardware was in place. The barred owls were all housed at the same location in the hospital in stainless steel cages (71 × 55 × 71 cm) with an astroturf substrate and astroturf-covered polyvinyl chloride perch. The cages, substrates, and perches were disinfected daily with a hydrogen peroxide disinfectant (Rescue™, Virox Technologies, Oakville, Ontario, Canada). They were also fed frozen–thawed mice equivalent to approximately 10% of their body weight once daily. The mice were all from the same source (RodentPro, Inglefield, IN, USA). After the birds’ fractures were confirmed to be healed, they were transferred to rehabilitation mews (15.2 × 3 × 5.5 m) constructed of wood slats (2.54 × 5.08 cm) and with a limestone gravel substrate for their pre-release flight training. Perches were constructed from treated lumber and covered with astroturf or rope. The same disinfection protocol was used on perch surfaces.

The sampling fine-tipped swab, provided by a commercial laboratory (MiDOG LLC, Tustin, CA, USA), was introduced into the cloaca and gently rolled ten times towards the coprodeum to include fecal material, if possible, while the cloaca was held open with sterilized Kelly forceps to avoid contamination from peri-cloacal microorganisms. This method was conducted on three cadavers to ensure consistent sample collection. To compare cloacal and fecal samples, paired samples from a single NoAbs bird were also collected and included in the analysis. The swab was then placed into a collection tube containing commercial buffer (MiDOG LLC) and shipped at ambient temperature. The samples were collected at the time of the bird’s admission to the hospital and prior to release, which was between 6 and 12 weeks from the presentation. The only other treatments allowed for the patients were fluid therapy and analgesics. Subcutaneous fluids (Lactated Ringer’s solution, Abbott Labs, North Chicago, IL, USA) were provided subcutaneously over the first 24 h to correct any deficits and supply maintenance fluids (100 mL/kg/day). After 24 h, maintenance fluids were continued as needed for the case. Analgesics were provided prior to surgery and post-surgery based on the patient’s response to therapy and clinician preference. Meloxicam (2 mg/kg q12–24 h; Boehringer Ingelheim, St Joseph, MO, USA) and hydromorphone (0.5 mg/kg q12 h; Hikma Pharmaceuticals, Berkeley Heights, NJ, USA) were the only analgesics used for the owls.

### 2.2. Microbiome Analysis

Next-generation sequencing was conducted targeting the bacterial microbiome using the V1-V3 region of the 16S rRNA gene (MiDOG LLC). The genomic DNA was purified using the ZymoBIOMICS-96 DNA kit (Cat. No. D4304, Zymo Research Corp., Irvine, CA, USA). Sample library preparation and data analysis for bacterial profiling were performed using the Quick-16S NGS Library Prep Kit (Cat. No. D6400, Zymo Research Corp.), with minor modifications. Libraries were sequenced using an Illumina HiSeq 1500 sequencer (Illumina, San Diego, CA, USA). Reads were filtered through Dada2 (R package version 3.4) to remove reads with low-quality, host-derived, and chimeric sequences before analysis. Taxonomy classification was performed using Centrifuge software (version 1.0.4; Johns Hopkins University, Baltimore, MD, USA) in combination with a custom reference database (version 24; Zymo Research) curated, in part, from draft or complete genomic sequences available from GenBank (National Center for Biotechnology Information, Bethesda, MD 20894, USA), as well as other publicly available bacterial taxonomic databases including the SILVA database. A sequence similarity of 97% was used to assign species identification, whereas operational taxonomic units (OTUs) with <97% sequence similarity were assigned to the genus level. Internal controls were used to ensure the accuracy and cleanliness of the data and to control for potential contamination of the equipment or sequencing buffers. Negative controls were also run for the storage buffer (catalog number R1100-50; DNA/RNA Shield), which was lysed, extracted, library-prepped, and sequenced in parallel with the experimental samples, and for the library preparation, less than 10% of low-count and low-variance features were filtered out, and the data were normalized using Total Sum Scaling.

Two sets of comparisons were performed in the study. The first comparison included serial samples (within-subject design) from NoAbs owls at presentation (baseline) and prior to their release (n = 4). The second comparison (between-subject design) included samples from Abs birds (n = 4) and NoAbs birds (n = 8) prior to their release. The 95% binomial confidence intervals (CIs) using the Wilson method were calculated for the bacterial proportions. Alpha diversity metrics were calculated using Chao1, Shannon, and Simpson’s D indices using the Wilcoxon matched-pairs signed-rank test or the Mann–Whitney test, depending on the dataset. Beta diversity was examined using a principal coordinate analysis as the distance-based ordination technique, Bray–Curtis dissimilarity as the distance method, and PERMANOVA as the statistical method. Linear discriminant analysis effect size (LEfSe) was conducted with a score threshold of 2.0. Additional analysis was performed using the MicrobiomeAnalyst R package (https://www.microbiomeanalyst.ca) [16] and GraphPad Prism V9.0 (GraphPad Software, San Diego, CA, USA). A *p*-value < 0.05 was used to determine statistical significance.

### 2.3. Antimicrobial Resistance Gene Analysis

The AMR genes were detected using a proprietary sequencing method capable of identifying a minimum of 80 AMR genes by the lab (MiDOG LLC). This method utilized an amplicon-based sequencing technique with proprietary PCR primers designed from AMR gene sequences available in the National Center for Biotechnology Information (NCBI) database. The specificity and reproducibility of the results were confirmed through cross-referencing with the Comprehensive Antibiotic Resistance Database (CARD).

## 3. Results

### 3.1. Treatment

Treatment durations for the owls in the Abs and NoAbs groups for the analgesics can be found in Table 1. The Mann–Whitney U test revealed that there were no statistical differences in the duration of the meloxicam and hydromorphone between Abs and NoAbs groups (*p* = 0.18, 0.39, respectively). All individuals included in the study successfully completed the treatment without any other complications. Following 8–24 weeks of rehabilitation, all birds were released within one to two weeks of pre-release samplings.

### 3.2. Bacterial Composition of the Gut Microbiome

Two samples with <10,000 reads and <10 species richness were excluded from the analysis. The final sample information included in the analysis is shown in Table 2. After filtering, 90% of the total sequencing reads were retained. The remaining samples had sequencing depths ranging from 16,844 to 34,880 reads (mean ± SD: 27,488.3 ± 5865.4), which were considered sufficient for downstream analysis. Raw read data is provided in Table S3. All rarefaction curves reached a plateau, confirming that the sequencing depth was adequate (Figure S1).

**Figure 1:**
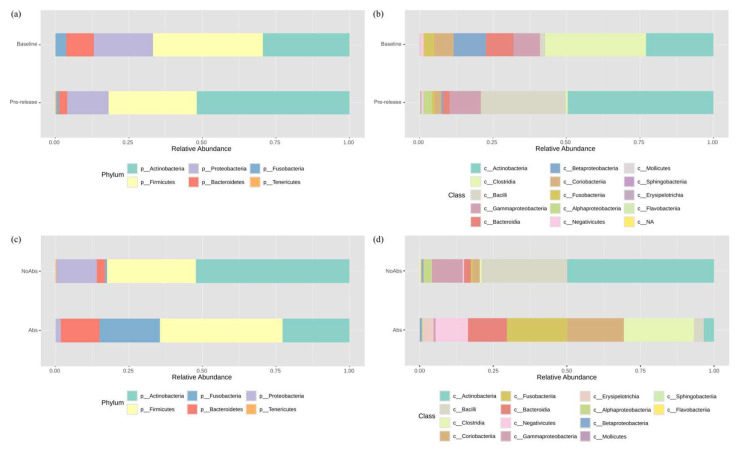
Taxonomic bacterial composition of barred owls on 16S rRNA sequencing. Composition is shown at the (**a**) phylum and (**b**) class levels for owls that did not receive antibiotics at the baseline (n = 4) and in the pre-release samples (n = 7). Additionally, bacterial composition at the (**c**) phylum and (**d**) class levels is presented for owls that did not receive antibiotics (NoAbs; the same samples of the pre-release group in [a] and [b], n = 7) and those that received antibiotics (Abs, n = 4) prior to release.

The taxonomic bacterial composition of the baseline and the pre-release samples from NoAbs barred owls is shown in Figure 1 and Table S1. In order to provide an overview of the bacterial community, all available samples were included in this analysis (n = 4 in the baseline; n = 7 in the pre-release group: 3 from the original baseline, only 1 sampled at the baseline, and only 3 sampled at pre-release), regardless of overlapping individuals. Statistical comparisons were not performed due to the lack of matched samples between the groups; instead, only overlapping birds (n = 3) were used for subsequent analyses.

The most abundant phyla in the baseline samples were Firmicutes (39.5%, 95% CI: 10–79.2%), Actinobacteria (28.5%, 95% CI: 5.7–72.3%), Proteobacteria (19.3%, 95% CI: 2.9–65.8%), Bacteroidetes (9.1%, 95% CI: 0.7–57.5%), and Fusobacteria (3.5%, 95% CI: 0.1–52.4%). At the class level, Clostridia (36.8%, 95% CI: 8.9–77.6%), Actinobacteria (22.2%, 95% CI: 3.7–67.9%), Betaproteobacteria (10.6%, 95% CI: 1–58.8%), Bacteroidia (9.1%, 95% CI: 0.7–57.5%), Gammaproteobacteria (8.5%, 95% CI: 0.6–57%), Coriobacteriia (6.3%, 95% CI: 0.4–55%), and Fusobacteriia (3.5%, 95% CI: 0.1–52.4%) were the most abundant.

The pre-release samples in NoAbs owls were dominated by Actinobacteria (52.1%, 95% CI: 21.6–81.1%), Firmicutes (29.8%, 95% CI: 8.8–65.1%), Proteobacteria (14%, 95% CI: 2.5–51%), and Bacteroides (2.8%, 95% CI: 0.1–38.9%). Regarding the class level, Actinobacteria (49.8%, 95% CI: 20.1–79.6%) accounted for almost half of the bacterial community, followed by Bacilli (28.9%, 95% CI: 8.4–64.4%), Gammaproteobacteria (10.6%, 95% CI: 1.5–47.6%), Alphaproteobacteria (3%, 95% CI: 0.1–39.2%), Coriobacteriia (2.3%, 95% CI: 0.1–38.3%), and Bacteroidia (2.3%, 95% CI: 0.1–38.3%).

The most dominant phyla in the pre-release Abs owls were Firmicutes (40.7%, 95% CI: 10.6–79.9%), Actinobacteria (22.9%, 95% CI: 3.9–68.4%), Fusobacteria (20.6%, 95% CI: 3.2–66.8%), and Bacteroidetes (14%, 95% CI: 1.6–61.7%). At the class level, Clostridia (23.9%, 95% CI: 4.2–69.2%), Fusobacteriia (20.6%, 95% CI: 3.2–66.8%), Coriobacteriia (19.3%, 95% CI: 2.9–65.8%), Bacteroidia (14%, 95% CI: 1.6–61.7%), Negativicutes (10.8%, 95% CI: 1–59%), Actinobacteria (3.5%, 95% CI: 0.1–52.4%), and Bacilli (3.3%, 95% CI: 0.1–52.2%) were identified.

Firmicutes and Actinobacteria remained dominant across all groups; however, Actinobacteria exhibited a lower proportion in the pre-release Abs samples, with a downward-shifted CI range. Proteobacteria were exclusively detected in the NoAbs group, including both the baseline and the pre-release, suggesting a potential suppressive effect of antibiotic exposure. Although the baseline samples of the Abs group were not available for analysis, Fusobacteria, which were initially present at a low proportion in the baseline samples and absent in the pre-release NoAbs samples, emerged at a higher proportion in the pre-release Abs samples, indicating a possible increase with antibiotic exposure. Actinobacteria and Bacilli were distinctly more abundant in the pre-release NoAbs group, as indicated by their 95% CIs, which showed no overlap between the groups. Clostridia were not detected (<1%) in the pre-release NoAbs group. The CIs of many phyla and classes overlapped, likely due to the small sample size.

### 3.3. Diversity Analysis

There was no significant difference in alpha diversity (Chao1, Shannon, or Simpson indices, all *p* > 0.99) for the baseline and the pre-release samples from the NoAbs owls. Likewise, beta diversity (Bray–Curtis index) did not differ between the two groups (*p* = 0.5).

**Figure 2:**
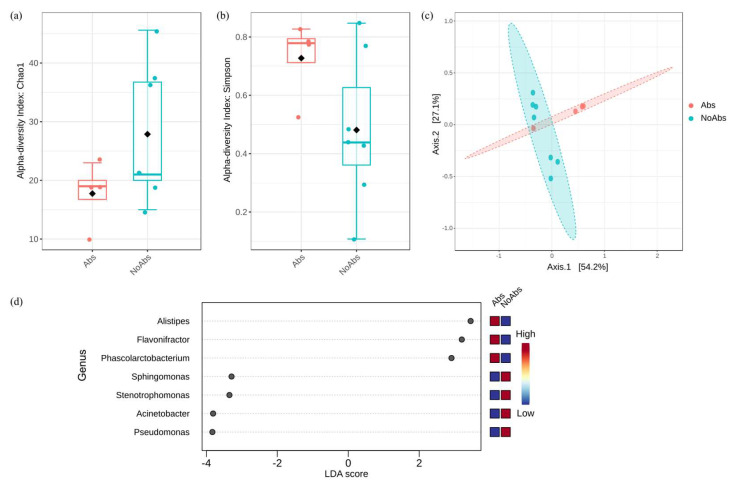
Comparison of the bacteria identified in antibiotic-treated (Abs) and non-antibiotic-treated (NoAbs) owls prior to release. Alpha diversity metrics, including (**a**) Chao1 and (**b**) Simpson indices, indicated significantly higher diversity in the NoAbs group. (**c**) Principal coordinate analysis (PCoA) based on the Bray–Curtis index at the class level confirmed a significant difference between the two groups. (**d**) Linear discriminant analysis effect size (LEfSe) identified differentially abundant genera.

In contrast, when comparing the pre-release samples of NoAbs and Abs owls, the Chao1 index was significantly higher in the NoAbs group (*p* = 0.041), indicating greater species richness. Similarly, Simpson’s D index was significantly higher in the Abs group (*p* = 0.034), suggesting that bacterial communities in the NoAbs group were more diverse and evenly distributed too (Figure 2). Beta diversity, assessed at the feature level, showed a marginally significant trend (*p* = 0.052) and reached significance at the class level (*p* = 0.035). LEfSe identified significantly different genera between the groups. *Alistipes* (LDA score = 3.45), *Flavonifractor* (3.18), and *Phascolarctobacterium* (2.92) were more abundant in Abs birds, while *Pseudomonas* (–3.84), *Acinetobacter* (–3.83), *Stenotrophomonas* (–3.35), and *Sphingomonas* (–3.29) were more abundant in NoAbs birds.

### 3.4. Antimicrobial Resistance Detection

No sample was excluded from the AMR gene analysis (n = 17). All rarefaction curves reached a plateau, confirming that the sequencing depth was sufficient. Table 3 shows the AMR genes detected, resistance mechanisms, and the number of genes detected per group.

Among the baseline samples representing the wild microbiome (n = 5), AMR genes conferring resistance to aminoglycosides, carbapenems, cephalosporins, cephamycins, lincosamides, macrolides, monobactams, phenicols, streptogramins, sulfonamides, and tetracyclines were detected. In the pre-release NoAbs samples from (n = 8), genes resistant to aminoglycosides, carbapenems, cephalosporins, cephamycins, fluoroquinolones, lincosamides, macrolides, monobactams, penams, phenicols, streptogramins, sulfonamides, and tetracyclines were found. Pre-release Abs samples (n = 4) contained resistant genes against aminoglycosides, fluoroquinolones, penams, phenicols, streptogramins, macrolides, lincosamides, sulfonamides, and tetracyclines.

When only the overlapping NoAbs individuals (n = 4) were considered, the number of detected AMR genes did not increase over hospitalization. LEfSe analysis revealed that aminoglycoside phosphotransferase (LDA score = −2.28) was enriched in the baseline, whereas lincosamide nucleotidyltransferase (3.27) was notably more abundant in the pre-release group. At the pre-release stage, tetracycline resistance genes were exclusively detected in the Abs group, but they were absent in the NoAbs group.

## 4. Discussion

This study characterized the cloacal bacterial composition of barred owls that were recently rescued and those hospitalized for over two months. Firmicutes and Actinobacteria were the dominant phyla, which was a consistent trend across all barred owl samples, regardless of hospitalization period. These phyla likely dominate due to the owls’ carnivorous diet, which requires efficient protein and fat metabolism and antimicrobial protection in the gut [17,18,19]. Firmicutes have similarly dominated the gut microbiome in other raptors [19,20], including barn owls (*Tyto alba*) [21], western screech owls (*Megascops kennicottii*), and whiskered screech owls (*Megascops trichopsis*) [22]. Among Firmicutes, Clostridia predominated—a class known for its role in protein metabolism; the production of short-chain fatty acids (SCFAs), particularly butyrate; and resistance against newly introduced organisms as part of the commensal gut microbiota [17,23,24]. These findings align with studies on scavenging vultures where Clostridia were found in high proportions and contributed to protein metabolism and tolerance to the harsh gut environment associated with a scavenger diet [25,26,27]. The proportion of Bacteroidetes was lower than that observed in herbivorous birds, a pattern commonly seen in carnivores with a high Firmicutes-to-Bacteroidetes ratio.

Corynebacteriales predominated in 30% (7/23) of samples in which Actinobacteria constituted over 60% of the bacterial composition; however, they could not be identified to the family or genus level. This order encompasses environmental, commensal, and opportunistic species, some of which are known to colonize mucosal and cutaneous surfaces in vertebrates [28]. These bacteria have also been detected in cloacal samples from passerines [29,30] and lesser kestrels (*Falco naumanni*) [31]. Moreover, the lesser kestrel cloacal samples contained an overwhelmingly high proportion of Corynebacteriales, a finding that differed from their phylogenetic relative, the common kestrels (*Falco tinnunculus*), in which only 33% of examined feces had more than 20% of Actinobacteria [32]. The cloaca can harbor microbes not only from feces but also from sexually transmitted and environmentally derived bacteria [33]. While Corynebacteriales were likely derived from feces, their high prevalence in some samples suggests they may also be stable residents of the cloacal mucosa, potentially playing a role in mucosal homeostasis. Another possible explanation is that increased abundance may be linked to stress during hospitalization, as previous studies reported significantly higher levels in fecal samples from temporarily sedated common cranes (*Grus grus*) compared to naturally defecated samples [34] or in rescued raptors [19]. Still, the possibility that these compositions reflect barred owls’ normal variation cannot be excluded.

To date, it remains unclear whether fecal or intestinal samples best reflect the “true” gastrointestinal microbiome. Many studies have used excreted fecal samples for microbiome analysis in both mammals and birds, while cloacal swab samples have also been utilized in birds to minimize human interactions but obtain fresh, uncontaminated samples. A study comparing sampling methods in several bat species found that fecal samples retained more dietary signals, whereas intestinal tissue samples were better suited for studying host evolution [35]. A similar finding was reported in California condors (*Gymnogyps californianus*), having functional differences between fecal and cloacal microbiota [27]. In ostriches (*Struthio camelus*), neither fecal nor cloacal swabs accurately represented the microbiota of the ileum and cecum; however, cloacal swabs exhibited the strongest correlation with the colon’s microbiota that was best represented in the feces [36]. Another study conducted using zebra finches (*Taeniopygia guttata)* reported that cloacal samples resembled those of feces, which were representative of the large intestinal microbes [37]. They suggested this difference might originate from the small diameter of the colon in zebra finches compared to ostriches. Given these findings, cloacal samples can be used to infer gut bacterial composition if sampling conditions remain consistent. Since minimizing human contact is essential in wildlife rehabilitation, we opted for cloacal sampling rather than checking for feces in real time and aimed to include samples representative of the colon.

We compared samples from a single cloacal swab and fresh feces collected at the same time point from the same bird (NoAbs; Table S2 and Figure S2). Due to the single sample, statistical analysis was not performed; however, notable differences were observed in bacterial composition, such as a 60% increase in Firmicutes, an 86% decrease in Actinobacteria, and a 36% decrease in Proteobacteria in the fecal sample. The main shifted genera were *Clostridium*, not-assigned Corynebacteriales, and *Escherichia*. These shifts may be attributed to the aerobic environment of the cloaca and the presence of a distinct mucosal microbial community, which was noted in the California condors and ostriches. Additionally, urine and uric acid passing through the cloaca can also affect microbiota. However, despite variations in relative abundance, alpha diversity indices and AMR gene compositions showed high similarity.

When comparing the within-NoAbs bird samples at initial admission and before release, there was no significant difference in bacterial taxa based on LEfSe analysis. Avian gut microbiota can be influenced by factors such as diet, health status, captivity stress, microbial environment, and habitat, even within the same species [38,39,40,41,42,43,44]. Phylogenetically related species often exhibit distinct microbiota compositions [45]. In humans and mice, orally ingested microbes typically appear in fecal samples within a day [46,47], and diet has been shown to strongly influence gut microbiota [38,39]. Nonetheless, our analysis revealed no significant changes in gut microbiome diversities following hospitalization for fracture repair, suggesting that short-term dietary shifts had a limited impact on these adult barred owls. This does align with a human study in which alpha diversity did not significantly change before, during, or after a period of strict animal-based diet consumption [47]. The absence of change is considered positive because it suggests the stress of being hospitalized did not significantly affect the birds’ fitness prior to release. Following the birds post-release would be essential to assess any potential long-term effects on their fitness.

Contrary to our results, other studies have found a decrease in gut microbiome diversity in birds under captivity status, even in the absence of antibiotic use [20,41]. The metabolic function of rehabilitated raptors was also found to decrease in these cases [20]. However, other research suggested that changes in bacterial composition do not always correlate to significant metabolic shifts because taxonomically different bacteria can perform similar functions [48]. This remains particularly unclear in birds because most microbiome–metabolism studies have focused on humans and mammals [49]. Moreover, studies on the intestinal microbiota of wild wood mice (*Apodemus sylvaticus*) suggest seasonal fluctuations due to dietary changes, while the gut microbiota of American red squirrels (*Tamiasciurus hudsonicus*) also exhibits temporal variation, supporting the idea that microbiome composition can naturally fluctuate [50,51]. Since most animal microbiome studies have analyzed momentary snapshots without accounting for natural variations, a more comprehensive approach targeting larger communities is needed. Our results, combined with a report that dietary changes in young piglets did not alter the gut microbiota for one month [38], indicate that adult barred owls appear to maintain their gut microbiota at a constant level, contrary to our concerns. Nonetheless, all birds exhibited some changes in their gut microbiota—marked by a reduction in Clostridiales and an increase in Bacillales. Further studies with larger sample sizes are needed to determine the relative contributions of prey-associated microbiota and environmental exposure to these changes. As a particularly large individual variability in carnivores has been reported, having more than 10 samples from the same species was recommended to represent the gut microbiota of that species reliably [52].

In contrast, the Abs group exhibited a significant reduction in bacterial richness and evenness compared to the NoAbs group. In addition, the Bray–Curtis distance analysis confirmed a significant shift between groups, highlighting the impact of antibiotic treatment. Broad-spectrum antibiotics can significantly disrupt approximately 30% of bacterial species, leading to a rapid decline in taxonomic richness, diversity, and evenness [53]. These shifts can lead the microbiome to function in a manner that mimics a diseased state. Our findings also revealed a greater than 30% decline in bacterial richness and evenness. The bacteria that were significantly more abundant in the NoAbs group were *Pseudomonas*, *Sphingomonas*, *Stenotrophomonas*, and *Acinetobacter*—widely distributed in the environment and also commonly found in hospital settings. The significant reduction in these taxa in the Abs group suggests that enrofloxacin, a fluoroquinolone antibiotic used during treatment, suppressed these opportunistic bacteria, altering microbial community dynamics. Given that both groups were exposed to the same diet and hospital environment, this shift can be attributed to antibiotic use rather than other external factors. In the Abs group, bacteria such as *Alistipes*, potentially related to the inflammatory intestinal environment, increased, whereas *Phascolarctobacterium*, bacteria with the SCFA-producing ability, also increased, suggesting a compensatory shift toward establishing a new balance.

Following antibiotic cessation in humans, the microbiome shows resilience but rarely fully returns to baseline. Even short-term antibiotic use can cause disruptions that persist for extended periods [54,55,56]. A study in mice reported that oral antibiotic administration reduced ‘colonization resistance’—the ability to eliminate newly introduced organisms—immediately after treatment, and this reduction remained significant throughout the antibiotic regimen [23]. After the discontinuation of the antibiotics, the susceptibility to external microbial invasion gradually decreased, and recovery was accelerated when the affected mice were housed with normal mice [46]. This finding is consistent with a study on dugongs (*Dugong dugon*), where 8 months later, individuals released back into the wild exhibited gut microbiota more similar to wild populations than to those of captive individuals, suggesting that wild foraging, natural environments, and interactions with wild colonies contributed to microbiome restoration [57]. Similarly, in our study, Abs owls still differed from NoAbs individuals at the time of release, which was 8–24 weeks past the discontinuation of the drug. Nevertheless, repopulation and microbial reassembly are expected to restore a healthier composition over time, a process that is likely to be accelerated through exposure to diverse microorganisms in the wild after release. Still, as observed in Tasmanian devils (*Sarcophilus harrisii*), where individuals shifted toward a wild microbiome post-release but retained certain captivity-acquired microbes [58], there remains a strong possibility that newly acquired bacteria and antimicrobial resistance genes will persist even after release. More studies on barred owls, and raptors in general, are needed to further measure this because of their roles at the top of the food web.

As hypothesized, AMR genes were detected in the majority of samples, regardless of hospitalization or antibiotic treatment, except for three samples collected at the time of release—two from NoAbs birds and one from an Abs bird. The detection of a wide variety of resistance genes against carbapenems, monobactams, fluoroquinolones, tetracyclines, macrolides, and aminoglycosides in baseline samples highlights the widespread presence of antimicrobial resistance in the environment, which is likely influenced by anthropogenic factors such as agricultural runoff or wastewater contamination. A study on migratory birds found that their gut microbiota harbored resistance genes against most major antibiotic classes used in clinical and agricultural settings, with tetracyclines, macrolide–lincosamide–streptogramin, and beta-lactams being the most prevalent [59].

When the same individuals (NoAbs) were serially measured, lincosamide resistance genes significantly increased before release. Although we did not analyze microbial composition in the hospital environment or the frozen–thawed mice provided as feed, our findings suggest that the hospitalization led to a resistome shift likely due to the common use of lincosamide antibiotics such as clindamycin in hospital settings. This finding aligns with previous research showing increased resistance in *E. coli* at the time of release in rehabilitated wild birds [20]. Environmental and dietary shifts during hospitalization may have altered the gut microbiota, reducing certain resistant taxa while allowing hospital-acquired strains to proliferate, possibly through competitive exclusion. However, even if resistant bacteria are present, they may not always be detected because of fluctuations in microbial strains and sampling limitations. Moreover, while the molecular detection of AMR genes provides valuable insights into resistance potential, the presence of genes does not necessarily indicate phenotypic resistance, as gene expression and additional mechanisms may influence actual AMR susceptibility [60]. Since the targeted PCR approach used for AMR gene detection was limited to known resistance genes, shotgun metagenomic sequencing might provide a broader approach for assessing AMR and its potential impact on the environment, despite its own limitations.

According to a recent study, enrofloxacin and amoxicillin–clavulanic acid were the most commonly used antibiotics in wildlife rehabilitation facilities in the United States [61]. Our study found that the tetWNW gene was significantly more abundant in the enrofloxacin-treated group compared to the control group. However, its presence in baseline samples suggests other contributing factors beyond treatment alone. Long-term studies on rehabilitated animals are necessary to better understand the impact of newly acquired or modified bacterial populations on the host and external environment. Vultures carrying various pathogenic genes that can affect both animals and humans underscore the importance of continued epidemiological surveillance from a One Health perspective [26]. Given these findings, it is crucial to establish long-term management plans that optimize patient care while minimizing risks to the environment, and public health should be prioritized.

This pilot study includes a few limitations. First, as the study was conducted on actual rescued patients, although group differences were of minimal concern, the population was unbalanced and opportunistic. While no statistical difference was found, the duration of drug administration was not uniform across individuals. Further studies should investigate whether prolonged treatment has a greater impact on microbiome shifts. Despite a power analysis, a change in bacterial composition was observed between baseline and pre-release samples in the NoAbs group. Therefore, larger studies are required to clarify this issue more adequately.

## 5. Conclusions

Our study confirmed that the gut microbiota of barred owls closely resembles that of previously reported raptors. Despite initial concerns, the non-antibiotic-treated group showed no significant changes in microbial diversity between admission and release, suggesting that the gut microbiota of adult owls remains stable during short-term rehabilitation. In contrast, a commonly used antibiotic in wildlife clinics significantly altered bacterial composition compared to the non-treated group. Additionally, antimicrobial-resistant bacteria emerged during hospitalization, even in owls that did not receive antibiotics. These findings represent an important first step in understanding how routine wildlife treatment and release may influence the environment and One Health. Future longitudinal studies with larger sample sizes are needed to build upon these results.

## Figures and Tables

**Table 1:** Duration of meloxicam and hydromorphone treatments in barred owls that were treated or not treated with enrofloxacin in this study (median [interquartile range, min-max]).

Drug	Antibiotic Treatment	Duration of Treatment
Meloxicam	Yes	11 (9–13, 9–14)
	No	5 (4–9, 0–19)
Hydromorphone	Yes	2 (0–4, 0–5)
	No	0 (0–6, 0–8)

**Table 2:** Sampling time points for the barred owls included for the two comparisons. The two subjects that are crossed out were excluded due to poor analytical quality. Circled subjects were used to measure the effect of hospitalization within individuals over time. Birds within the rectangles represent the non-antibiotic birds and antibiotic birds at the time of pre-release. Colored cells show the period of time antibiotics were used in 4 birds after surgery.

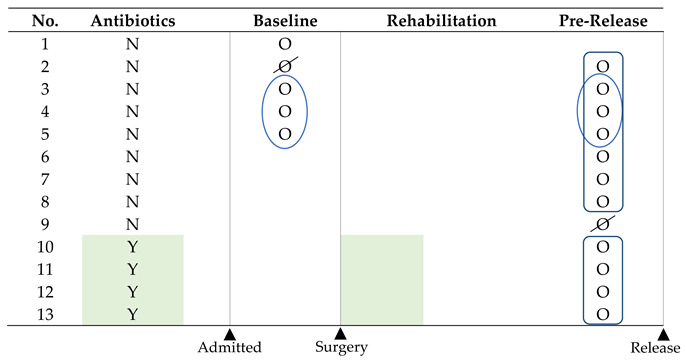

**Table 3:** Overview of antimicrobial-resistant (AMR) genes detected in cloacal samples of barred owls (total n = 17). The three categorized groups are baseline (n = 5), pre-release samples of NoAbs birds (PR-NoAbs, n = 8), and pre-release samples of Abs birds (PR-Abs, n = 4).

Against Antibiotics	AMR Gene	Resistance Mechanism (Enzyme)	Baseline	PR-NoAbs	PR-Abs
Number of owls			5	8	4
Aminoglycoside	AAC(3)-IIb	Aminoglycoside acetyltransferase	1	1	-
	aadA	Aminoglycoside nucleotidyltransferase	-	1	1
	APH(3′)-IIIa	Aminoglycoside phosphotransferase	1	-	-
	APH(3″)-Ib *	Aminoglycoside phosphotransferase	3	1	1
	APH(6)-Id	Aminoglycoside phosphotransferase	2	1	1
	ANT(4′)-Ib	Kanamycin nucleotidyltransferase	1	-	1
Beta-lactams	blaZ	Class A beta-lactamase	-	3	2
	mecA	Penicillin-binding protein 2a	1	1	-
Fluoroquinolone	gyrA	DNA gyrase, subunit A (mutated)	-	1	1
MLS: macrolide, lincosamide, streptogramin	ermB	Ribosomal methylase	2	-	1
	ermC	23S rRNA methyltransferase	1	1	-
	ermX	Ribosomal RNA methyltransferase	1	2	-
	lnuA *	Lincosamide nucleotidyltransferase	1	3	1
	mphC	Macrolide phosphotransferase	1	-	-
	mphD	Macrolide phosphotransferase	1	1	-
	msrA	ABC-F ribosomal protection protein	-	1	-
	msrD	ABC-F ribosomal protection protein	1	-	-
Phenicol	cat	Chloramphenicol acetyltransferase	-	1	-
	cmx	Chloramphenicol exporter	1	1	1
Sulfonamide	sul1	Dihydropteroate synthase	1	1	1
	sul2	Dihydropteroate synthase	1	2	1
Tetracycline	tetC	Tetracycline efflux pump	1	-	-
	tetK	Tetracycline efflux pump	1	-	-
	tetL	Tetracycline efflux pump	1	-	-
	tetWNW	Ribosomal protection protein	2	-	2
Total			25	22	15

* LEfSe analysis of the same four NoAbs individual showed that APH(3″)-Ib was significantly enriched in the baseline, and lnuA was significantly enriched in the pre-release samples.

## Data Availability

The original datasets presented in the study are included in the article/Supplementary Materials; further inquiries can be directed to the corresponding author.

## Supplementary Materials

The following supporting information can be downloaded at https://www.mdpi.com/article/10.3390/ani15111643/s1: Figure S1: Rarefaction curves of all samples included in the study. Figure S2: Taxonomic bacterial composition of a fecal and a cloacal sample in the same barred owl. Table S1: Bacterial taxonomy of the baseline and pre-release samples identified in this study. Table S2: Bacterial taxonomy of the cloacal and fecal samples. Table S3: Read counts of bacterial taxa across all samples.
