# Supplementary figures and images for: A Preliminary Investigation of the Gastrointestinal Bacterial Microbiomes of Barred Owls (Strix varia) Admitted to a Wildlife Hospital
Source: Animals (Basel). 2025 Jun 3;15(11):1643. doi: 10.3390/ani15111643 (PMC12153569; doi:10.3390/ani15111643)

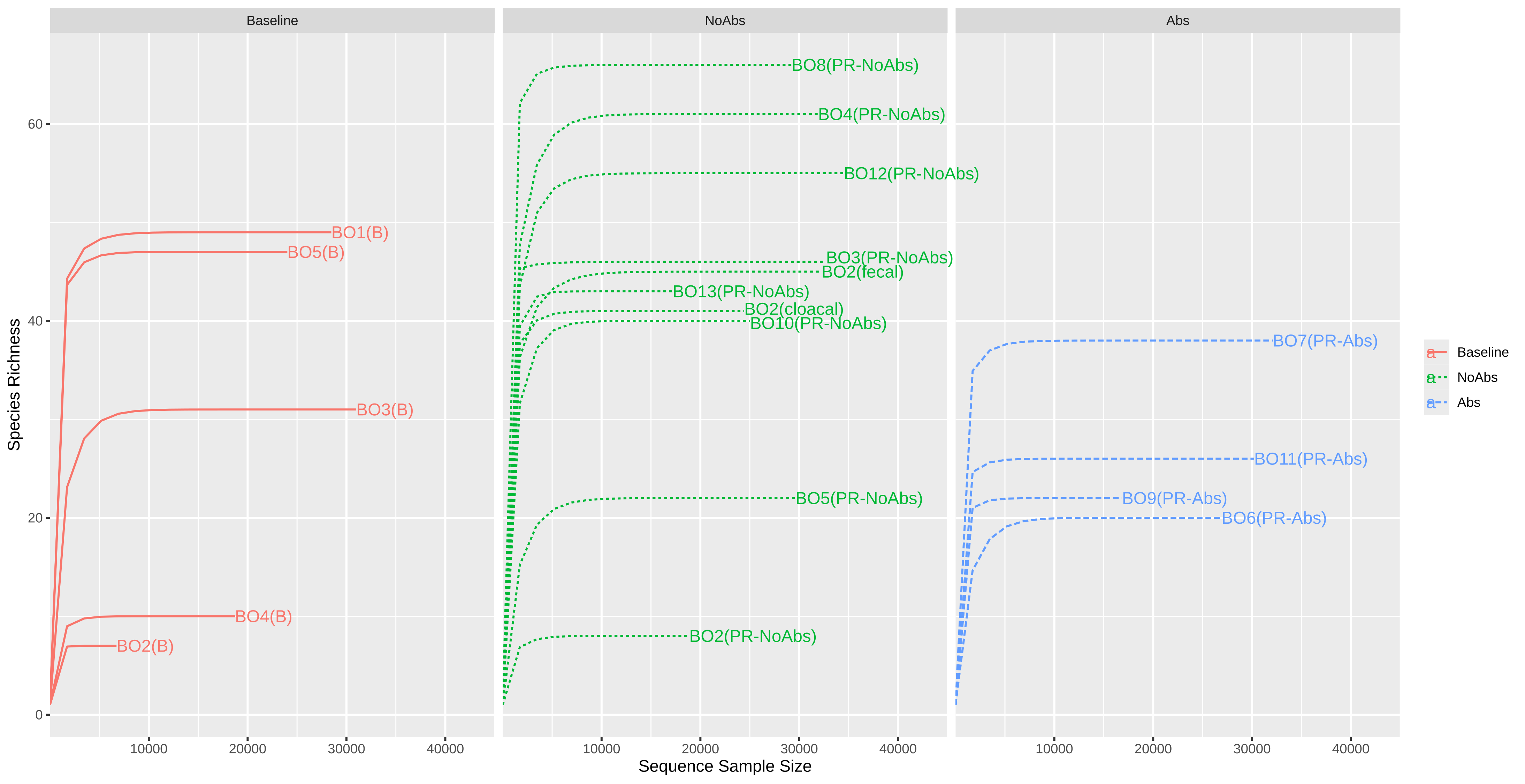

Supplement: Supplementary file 1 [file animals-15-01643-s001.zip › Supplement Figure S1.png]

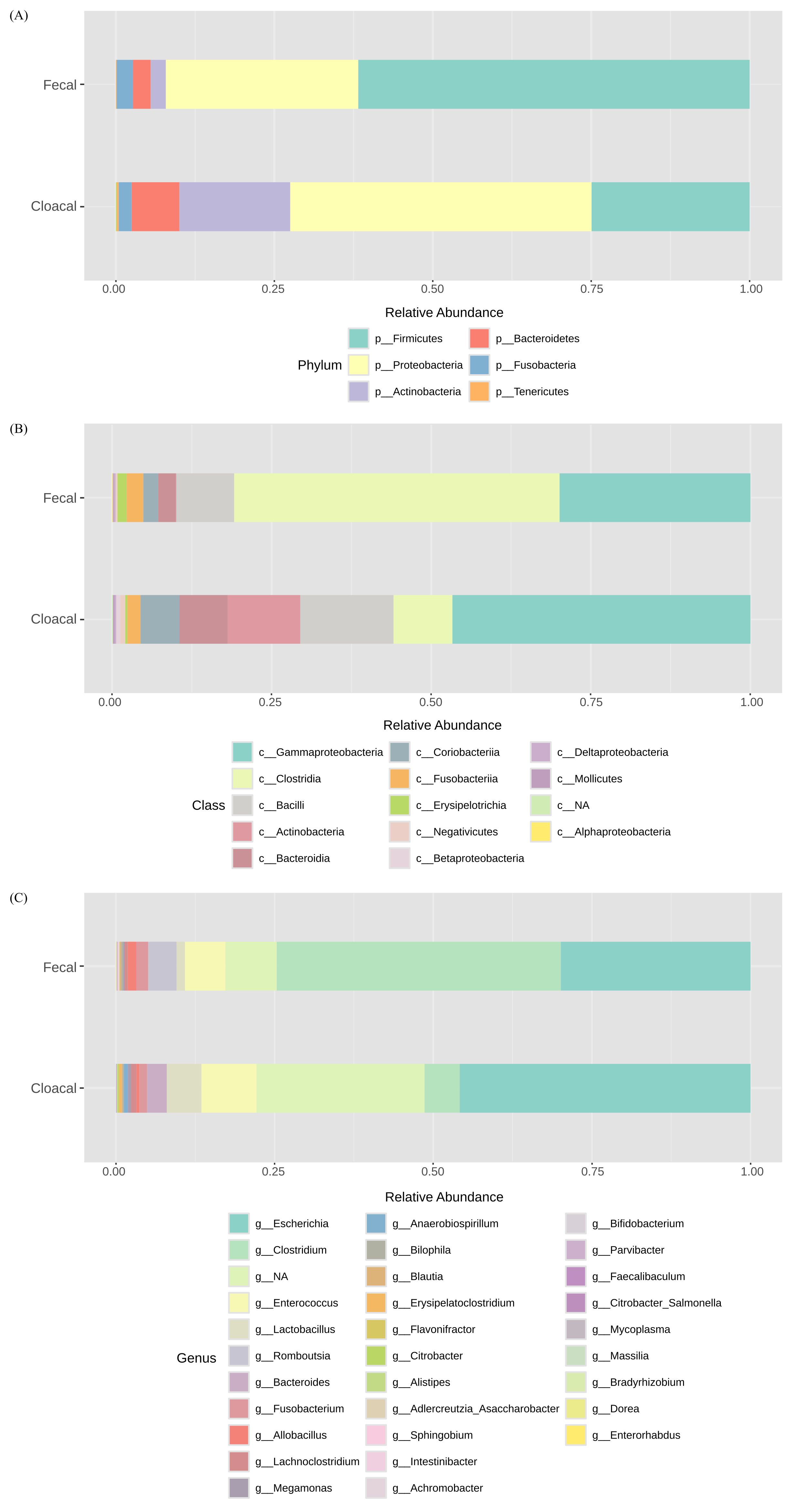

Supplement: Supplementary file 1 [file animals-15-01643-s001.zip › Supplement Figure S2.png]
